# Supplementary material for: Content-rich biological network constructed by mining PubMed abstracts
Source: BMC Bioinformatics. 2004 Oct 8;5:147. doi: 10.1186/1471-2105-5-147 (PMC528731; doi:10.1186/1471-2105-5-147)
Supplement: Additional File 5 — The original Chilibot query results of the term "long-term potentiation (LTP)" and 22 other terms, limiting the latest references analyzed to the years 1990, 1995, 2000, and 2004. [file 1471-2105-5-147-S5.bz2 › chilibotAdditionalFile5/ltp2000/html/PI-3K_PKC.html]

 


 **PI-3K** and **PKC** 
  
Found 132 abstracts in PubMed,  **30 abstracts were retrieved and analyzed**.  


---

 Search Google  |
 PDF files only 
|  EDU domain only 

---

**Interactive relationship** (e.g. stimulation, inhibition, etc)

- We found that LPS activates PI 3 kinase  [ **PI-3K** ]  and causes the formation of a  **PKC**  zeta PI 3 kinase  [ **PI-3K** ]  dependent kinase complex.  Ref: 11035106 J Immunol, 2000
- These data implicate the PI 3 kinase  [ **PI-3K** ]  pathway as an integral part of the LPS induced  **PKC**  zeta activation.  Ref: 11035106 J Immunol, 2000
- Similarly, in adipocytes of nondiabetic rats, rosiglitazone provoked increases in insulin stimulated 2 deoxyglucose uptake,  **PKC**  zeta lambda enzyme activity and phosphorylation of both threonine 410 activation loop and threonine 560 autophosphorylation sites in  **PKC**  zeta, but had no effect on PI 3 kinase  [ **PI-3K** ]  activation or PKB activation phosphorylation.  Ref: 10749857 J Biol Chem, 2000
- Rosiglitazone completely reversed defects in insulin stimulated 2 deoxyglucose uptake, PKCzeta lambda enzyme activity and  **PKC**  zeta threonine 410 phosphorylation, but had no effect on PI 3 kinase  [ **PI-3K** ]  activation or PKB activation phosphorylation in GK adipocytes.  Ref: 10749857 J Biol Chem, 2000
- Thus PKCzeta, and potentially other  **PKC**  isoenzymes, regulate growth factor mediated Akt phosphorylation and activation, which is consistent with a generalized role for PKCzeta in limiting growth factor signalling through the PI 3K  [ **PI-3K** ]  Akt pathway.  Ref: 11085941 Biochem J, 2000
- The effects of  **PKC**  on Akt were transmitted through the PI 3K  [ **PI-3K** ]  cascade as indicated by changes in p70 s6 kinase p70 s6k phosphorylation.  Ref: 11085941 Biochem J, 2000
- Use of inhibitors revealed that the betagamma independent pathway required activation of PI 3 kinases and was  **PKC**  independent In contrast, the betagamma dependent pathway involved activation of phospholipase C beta and  **PKC** , but was PI 3 kinase  [ **PI-3K** ]  independent.  Ref: 11078874 FEBS Lett, 2000
- Both Akt PKB, a serine threonine kinase with a PH domain, and atypical  **PKC**   **PKC**  zeta,  **PKC**  lambda have been implicated as downstream effectors of PI3 kinase  [ **PI-3K** ] .  Ref: 10707548 Nippon Rinsho, 2000
- **PKC**  zeta has been shown in other systems to be phosphorylated by phosphatidylinositol PI 3 kinase  [ **PI-3K** ]  dependent kinase.  Ref: 11035106 J Immunol, 2000
- Functional PI 3K  [ **PI-3K** ]  was required for the  **PKC**  inhibitors to increase Akt phosphorylation and activation, potentially owing to the activation of specific  **PKC**  isoforms by PI 3K  [ **PI-3K** ] .  Ref: 11085941 Biochem J, 2000

**Parallel relationship** (e.g. studied together, co-existance, homology, etc.)

- HGF signaling appears to involve activation of both  **PKC**  and PI3 kinase  [ **PI-3K** ] , inducing MAPK phosphorylation that is critical for migration and growth.  Ref: 10845613 Invest Ophthalmol Vis Sci, 2000
- Protein kinase C  **PKC** , MAPK, and PI3 kinase  [ **PI-3K** ]  involvement were evaluated using selective inhibitors and activity assays.  Ref: 10845613 Invest Ophthalmol Vis Sci, 2000
- We asked whether the inhibitory effect of EGF on secretion is due to activation of specific isoforms of protein kinase C  **PKC**  by PI 3 kinase  [ **PI-3K** ] .  Ref: 10801833 J Biol Chem, 2000
- The effect of ET 1 on glucose uptake was not inhibited by inhibitors for protein kinase C  **PKC** , protein kinase A PKA and phosphatidylinositol 3 kinase PI3 kinase  [ **PI-3K** ] .  Ref: 11078371 J Cardiovasc Pharmacol, 2000
- PC induced phosphorylation of protein kinase B and translocation of  **PKC**  epsilon, and it increased NO production, and these effects were blocked by WM, which suggests a role for PI3 kinase  [ **PI-3K** ]  in PC upstream of  **PKC**  and NO.  Ref: 10948065 Circ Res, 2000
- In adipocytes, insulin induced DG  **PKC**  signalling, PI 3 kinase  [ **PI-3K** ]  activation and  **PKC**  zeta translocation from cytosol to the membrane were suppressed when treated with PTX, despite no changes in 125I insulin specific binding and insulin receptor tyrosine kinase activity.  Ref: 10781929 Cell Signal, 2000
- We examined the effect of PTX on insulin induced glucose uptake, diacylglycerol DG protein kinase C  **PKC**  signalling, phosphatidylinositol PI 3 kinase  [ **PI-3K** ]  and  **PKC**  zeta activation and insulin induced tyrosine phosphorylation of Gialpha to clarify the role of G protein for insulin mediated signal transduction mechanism in rat adipocytes and soleus muscles.  Ref: 10781929 Cell Signal, 2000
- The present study is designed to test whether phosphatidylinositol 3 kinase PI3 kinase  [ **PI-3K** ]  has a role in the signaling pathway in ischemic preconditioning PC and whether it is proximal or distal to protein kinase C  **PKC** .  Ref: 10948065 Circ Res, 2000
- Inhibitors of tyrosine kinase and  **PKC** ... attenuated the insulin neurotoxicity.  Ref: 10690636 IUBMB Life, 1999
- Our results suggest that downstream of PI 3 kinase  [ **PI-3K** ] , insulin signalling diverges into at least two independent pathways through Akt PKB and  **PKC**  zeta, the  **PKC**  zeta pathway contributing to glucose transport induced by insulin in fetal brown adipocytes.  Ref: 10781824 FEBS Lett, 2000
- The activation of ERK1 and ERK2 by LPA was  **PKC**  and phosphatidylinositol 3 kinase PI 3 kinase  [ **PI-3K** ]  dependent.  Ref: 10704947 Biochem Pharmacol, 2000
- Combined  **PKC**  and PI3 kinase  [ **PI-3K** ]  inhibition was additive P <.  Ref: 10845613 Invest Ophthalmol Vis Sci, 2000
- However, AVP induced activation of RSK2, a downstream substrate of ERK1 and ERK2, was  **PKC**  dependent and PI 3 kinase  [ **PI-3K** ]  independent.  Ref: 10704947 Biochem Pharmacol, 2000
- Conversely, the inhibitor of PI3 kinase  [ **PI-3K** ]  but not  **PKC**  reversed the antiapoptotic effect of insulin.  Ref: 10690636 IUBMB Life, 1999
- HGF induced BREC growth was suppressed by PI3 kinase  [ **PI-3K** ] ,  **PKC** , or MAPK inhibition all P <.  Ref: 10845613 Invest Ophthalmol Vis Sci, 2000
- the obtained data suggest that an activation of alpha1A adrenoceptors, presence in WAT, by agonist and or neurotransmitter may increase the glucose uptake via PLC  **PKC**  pathway and the activation of PI 3 kinase  [ **PI-3K** ] .  Ref: 11111846 Auton Neurosci, 2000
- The possible participation of phosphatidylinositol PI 3 kinase  [ **PI-3K** ] , p44 42 mitogen activated protein MAP kinases and protein kinase C  **PKC**  in staurosporine induced prostaglandin E 2 PGE 2 production was investigated pharmacologically in rat peritoneal macrophages.  Ref: 10734171 J Pharmacol Exp Ther, 2000
- These data suggest that VEGF can increase CTGF gene expression in bovine retinal capillary cells via KDR or Flt receptors and the activation of PI3 kinase  [ **PI-3K** ]  Akt pathway independently of  **PKC**  or Ras ERK pathway, possibly inducing the fibrosis observed in retinal neovascular diseases.  Ref: 11018037 J Biol Chem, 2000
- These results suggest that G protein regulates DG  **PKC**  signalling by binding of Gialpha 2 with GTP and PI 3 kinase  [ **PI-3K** ]   **PKC**  zeta signalling by releasing of Gbetagamma via dissociation of trimeric G protein after insulin receptor tyrosine phosphorylation in insulin sensitive tissues.  Ref: 10781929 Cell Signal, 2000
- At the level of Raf 1, neither  **PKC**  iota nor PI3 kinase  [ **PI-3K** ]  was required for activation.  Ref: 10848576 Mol Cell Biol, 2000
